# Robotically Assisted Single Anastomosis Duodenoileal Bypass after Previous Sleeve Gastrectomy Implementing High Valuable Technology for Complex Procedures

**DOI:** 10.1155/2015/586419

**Published:** 2015-09-30

**Authors:** Ramon Vilallonga, José Manuel Fort, Enric Caubet, Oscar Gonzalez, José Maria Balibrea, Andrea Ciudin, Manel Armengol

**Affiliations:** ^1^Endocrine, Metabolic and Bariatric Unit, General Surgery Department, Vall d'Hebron University Hospital, Center of Excellence for the EAC-BC, Passeig de la Vall d'Hebron 119-129, 08035 Barcelona, Spain; ^2^Endocrinology Department, Vall d'Hebron University Hospital, Center of Excellence for the EAC-BC, Passeig de la Vall d'Hebron 119-129, 08035 Barcelona, Spain

## Abstract

Staged bariatric procedures in high risk patients are a common used strategy for morbid obese patients nowadays. After previous sleeve gastrectomy, surgical treatments in order to complete weight loss or comorbidities improvements or resolutions are possible. One strategy is to perform a novel technique named SADI (single anastomosis duodenoileal bypass-sleeve). We present the technique for totally intracorporeal robotically assisted SADI using five ports and a liver retractor. We aim to see if the robotic technology offers more advantageous anastomosis and dissection obtained by the robotic approach in comparison to standard laparoscopy. The safety, feasibility, and reproducibility of a minimally invasive robotic surgical approach to complex abdominal operations such as SADI are discussed.

## 1. Introduction

Sleeve gastrectomy (SG) constitutes the first stage of the duodenal switch and also of the SADI-S (single anastomosis duodenoileal bypass-sleeve) procedure [1]. SADI-S is a novel bariatric procedure based on the principles of biliopancreatic diversion (BPD) [2, 3]. On the other hand, the development of robotic surgical platforms such as the da Vinci Surgical System (Intuitive Surgical, Sunnyvale, CA), introduced in the surgical practice, has gained popularity in different specialities, including bariatric surgery. With the implementation of the robotic technology in our bariatric department, we wanted to study the feasibility, learning curve, and outcomes in robot-assisted sleeve gastrectomy [4–6]. One of the major issues related to the robotic technology is to know if it offers more advantageous anastomosis and dissection obtained by the robotic approach in comparison to standard laparoscopy as some authors have already mentioned.

Herein, we intend to present the first three cases of robotic SADI bypass as a second stage using a five-port technique and a liver retractor.

## 2. Patients and Surgical Technique (Table 1)

Patients' records were reviewed according to the institutional ethical committee. All three patients had a previous laparoscopic SG. Decision was made to undergo a robotic SADI bypass after completing a preoperative care assessment. All patients had medical criteria for revisional surgery (staged surgery), including patients with an insufficient weight loss (excess weight loss <50% after 18 months). All preoperative data are shown in Table 1.

### 2.1. Surgical Technique

**Figure 1:**
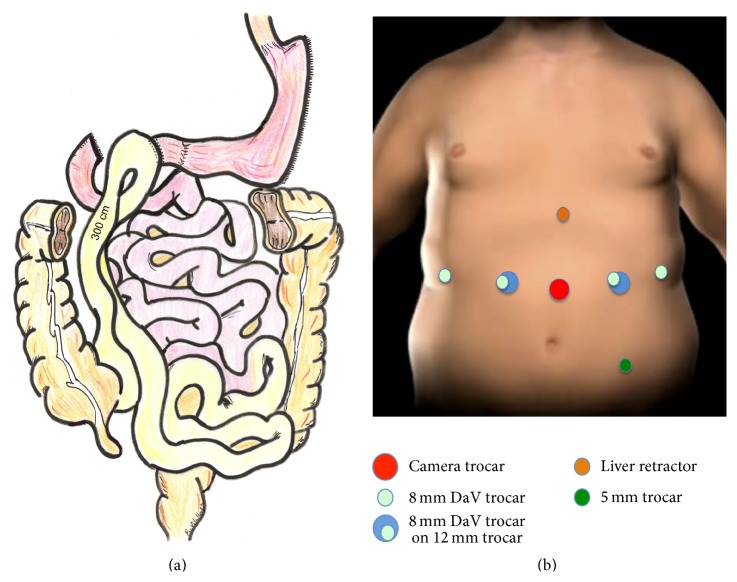
The SADI bypass includes a single duodenoileal anastomosis performed 300 cm from the ileocecal valve (a). Trocar placement according to the described technique (b).

SADI bypass includes a single duodenoileal anastomosis performed 300 cm from the ileocecal valve (Figure 1(a)).

### 2.2. Pneumoperitoneum and Trocar Placement

The pneumoperitoneum was achieved by a Veress needle inserted at the left hypochondrium. First trocar (camera trocar) was inserted slightly left of midline and 16 cm from the xyphoid. A 150 mm long trocar was used as the camera port (Xcel Trocar, Ethicon-Endosurgery, Cincinnati, OH, USA) that allowed the right connection with the robotic arm. All other trocars were inserted under direct vision. A 12 mm working port was inserted about 6 cm left of the previous trocar. The right 12 mm working port was positioned 6 cm from the camera port. Two 8 mm robotic trocars were placed on the anterior axillary lines. Finally, a Nathanson liver retractor was placed to elevate the left lateral segment. An extra 5 mm trocar was placed in the left iliac fossa. With this set-up in place, the left 8 mm robotic trocar could be used through the 12 mm trocar in a double-cannulation technique which allows deciding easily the best direction to perform the stapling of the duodenum (Figure 1(b)).

The bedside surgeon measures a 300 cm ileal loop laparoscopically. Then the robot was docked over the patient's head (covered with a head protection designed for this purpose). The da Vinci camera was locked in the midline trocar with placement of other instruments. The console surgeon inspected the previous sleeve and dissected all the inferior part of the previous sleeve identifying the old stapling line. All posterior adhesions to the top of the pancreas were freed. For this purpose, a grasper was used in the left hand and the da Vinci modified harmonic scalpel was installed in the right hand. The third da Vinci arm (the one placed on the left midaxillary line) was used with another pair of forceps in order to retract the previous sleeve and allow better exposition.

### 2.3. Sleeve Dissection, Duodenum Dissection and Section

**Figure 2:**
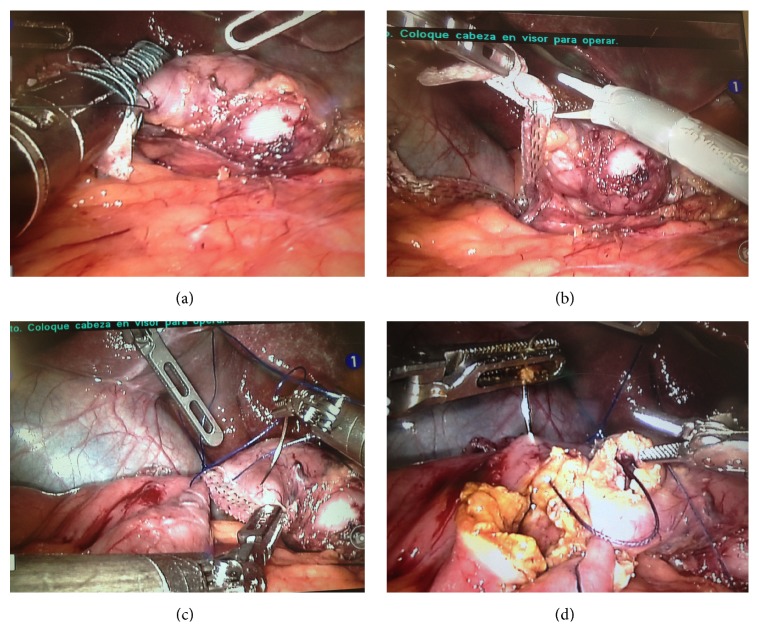
Complete transection of the duodenum (a). Excision of the buttress material reinforcement material located on the gastric part (b). Duodenoileal anastomosis, posterior polypropylene layer (c). Posterior continuous resorbable suture (d).

An important step of the SADI procedure is the complete dissection of the duodenum by identifying also the pyloric artery coming from the gastroduodenal artery. We preserved the artery. In this position, the robotic bedside cart does not give any difficulty to the anesthesiologist in order to place the bougie. Once the duodenal dissection was performed over 2 cm after pylorus, a laparoscopic stapler was used (Echelon 60 Endopath Stapler, Endoscopic Linear Cutter Straight, Ethicon-Endosurgery, Cincinnati, OH, USA) with a green cartridge including Seamguard buttress material reinforcement (Figure 2(a)). The complete transection of the duodenum was done.

**Figure 3:**
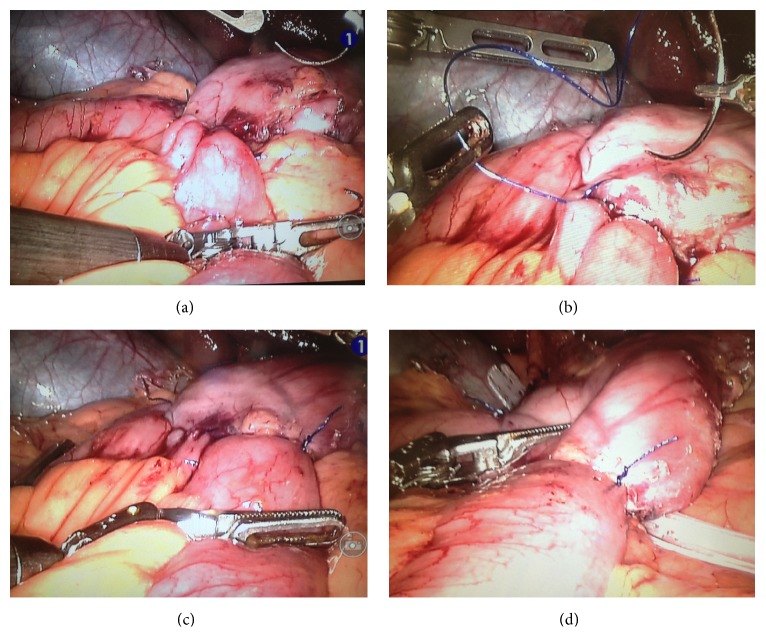
Duodenoileal anastomosis: anterior layer first with a vicryl continuous layer (a) and finally with a polypropylene 3/0 anterior closure (b). Anastomosis leak test (c). Drain placement (d).

Once transected, the duodenum was left in place and the buttress material reinforcement material located on the gastric part was cut by the use of robotic scissors (Figure 2(b)). The bedside surgeon introduced then a robotic needle holder in the left trocar. A totally robotic four-layer duodenoileal anastomosis was performed using polypropylene (3/0) (Prolene, Ethicon-Endosurgery). First a posterior polypropylene layer was made (Figure 2(c)); then the duodenum and the ileum were opened by the use of monopolar. A posterior continuous resorbable suture (vicryl 3/0 Ethicon-Endosurgery) was performed (Figure 2(d)) and then the anterior layer was constructed in similar fashion, first with a vicryl continuous layer (Figure 3(a)) and finally with a polypropylene 3/0 anterior closure (Figure 3(b)).

A methylene blue test is performed. For this maneuver, the console surgeon blocks the outlet at the level of the ileal loop in order to visualize the shape, the apparent volume, and any leak of the anastomosis (Figure 3(c)). A drain was left in place under the anastomosis and close to the duodenal stump (Figure 3(d)).

## 3. Results

The operative times of the three cases were 124, 174, and 138 min, respectively. There were no conversions and no mortality. There were no perioperative complications in the first 30 days. Patients were discharged after two days. The mortality rate was 0%. No barium swallow X-ray study was performed. Short-term follow-up data are shown in Table 1.

## 4. Discussion

SADI-S has been described as a novel bariatric operation based on the principles of biliopancreatic diversion (BPD) [2, 7]. This technique was proposed modifying a preexisting one to simplify the procedure, to decrease the potential complication rate, and to maintain or improve, if possible, the outcomes of the original biliopancreatic diversion [1].

The SADI-S procedure has been described as a single step or second-step procedure. In fact, primary robotic sleeve gastrectomy (RSG) was performed in patients with BMI over 50 Kg/m^2^ and after an initial weight loss [8]. In our previous report, we showed the feasibility of RSG and its potential use as a training model and developing a learning curve before undergoing more complex procedures [5, 7]. In a comparative study including 200 patients, no differences were found between the RSG group and the laparoscopic sleeve gastrectomy group [8].

In the second-stage SADI-S procedure, relevant issues must be mentioned. SADI-S as a second stage is considered a revisional bariatric procedure. The open approach remains the mainstay for these complex procedures, with use of the laparoscopic approach [9].

These procedures have an increased rate of complications related to bleeding, leaks, and even mortality [10, 11]. Hallowell et al. found a 2.5-fold increase in ICU stay and a 9-fold increase in leaks [12]. As in the mentioned series, a higher complexity during revisional cases included adhesions from the primary procedure, inflammation, and tissue changes. Complete robotic adhesiolysis must be performed in these patients, including liver dissection on previous sleeve gastrectomy and dissection of the antrum of the gastric sleeve. A clear dissection of the posterior sleeve, which is always adhered to the anterior part of the pancreas, must be performed avoiding pancreatic injury. The surgeon might consider placating the previous sleeve or resleeving the sleeve. Once this step is performed, a clear dissection of the duodenum including visualization and preservation of the gastroduodenal artery must be performed. Robotic da Vinci technology, including 3D vision, endowrist movement enables complex surgical movements for procedures like SADI-S as a first or second step [13, 14]. In the previous reports, we proposed robotic platform for SG which allows for quality dissection and handsewn suturing of the staple line [4]. A complete SADI-S procedure as a first step could be performed in our experience [8].

Another major issue is the connection of the duodenoileal anastomosis. The duodenoileal anastomosis is a complete four-layer hand-sewed anastomosis. In this type of anastomosis, robotic technology enhances surgeon's movements. As in another gastroenteroanastomosis performed for robotic gastric bypass, a major decrease of the leak rate can be achieved for the patient [15, 16]. Robotic technology eliminates the need for staplers in an environment where the thickness and quality of the tissues can be variable. For this purpose we believe and encourage performing the most complex procedures by the use of robotic technology in order to decrease the conversion to open surgery rate and intraoperative and postoperative major complications like bleeding and leak and perform more complex and demanding procedures.

SADI-S procedure, compared to DS, eliminates the Roux-en-Y reconstruction and includes a Billroth II-type one-loop duodenoileostomy instead [1, 2]. The Roux-en-Y intestinal reconstruction as a DS has already been described robotically [17]. In SADI-S procedure, the pylorus is preserved, thus avoiding the necessity to perform a Roux-en-Y diversion.

The elimination of one anastomosis should have further benefits: the reduction in the operation and anesthesia time, the reduction in the probability of postoperative leak. Furthermore, the use of the robotic system to perform this procedure facilitated and enhances the surgical work [13, 14]. The robotic platform can play a significant role in improving surgeon's ergonomics thus increasing the quality of the operation [14].

We believe that robotic technology will allow performing much more complex procedures, including revisional surgeries [13].

## 5. Conclusions

Robotic technology can be applied to perform SADI-S procedure. Standard technique can be applied in order to perform revisional surgery after failed or second-stage robotic SG. Larger case series and comparing to standard laparoscopic series will be required to analyze the impact of robotic surgery in more demanding procedures such as revisional bariatric surgery.

## Figures and Tables

**Table 1:** Data showing preoperative and postoperative comorbidities, weight, and BMI at time of sleeve gastrectomy surgery (SG), at the R-SADI, and at last follow-up.

Patient	Age/gender (years, M/F)	Weight and BMI at 1st SG (Kg/m^2^)	Comorbidities at 1st SG	Months between surgeries	Weight and BMI at R-SADI (Kg, Kg/m^2^)	Follow-up time (months)	Weight and BMI at last follow-up (Kg, Kg/m^2^)	Comorbidities at follow-up
1	34/M	49,87	None	14	91 34,3	9	75 28	None

2	59/M	57,66	*HTA* *OSAS + CPAP*	16	103,2 46,5	9	76 33	*HTA.*

3	56/M	51,35	Fibromyalgia DMT2 (insulin) HBP DLP Arthropathy	13	78,450 34,9	3	72 32	DMT2 (lower doses of insulin) HBP better

BMI: body mass index; M/F: male/female; SG: sleeve gastrectomy; R-SADI: robotic-single anastomosis duodenoileal bypass; GERD: gastroesophageal reflux disease; HBP: high blood pressure; DMT2: diabetes mellitus type 2; DLP: dyslipidemia.
